# Repair of cartilage defects in osteoarthritis rats with induced pluripotent stem cell derived chondrocytes

**DOI:** 10.1186/s12896-016-0306-5

**Published:** 2016-11-09

**Authors:** Yanxia Zhu, Xiaomin Wu, Yuhong Liang, Hongsheng Gu, Kedong Song, Xuenong Zou, Guangqian Zhou

**Affiliations:** 1 Shenzhen Key Laboratory for Anti-ageing and Regenerative Medicine, Health Science Center, Shenzhen University, Shenzhen, 518060 China; 2 Department of Spinal Surgery, The First Affiliated Hospital of Shenzhen University, Shenzhen, 518060 China; 3 State Key Laboratory of Fine Chemicals, Dalian R&D Center for Stem Cell and Tissue Engineering, Dalian University of Technology, Dalian, 116024 China; 4 Department of Spinal Surgery, Orthopaedic Research Institute, Huangpu Division, The First Affiliated Hospital of Sun Yat-sen University, Guangzhou, 510080 China

**Keywords:** iPSC, Chondrocyte, Osteoarthritis, Differentiation, Transplantation

## Abstract

**Background:**

The incapacity of articular cartilage (AC) for self-repair after damage ultimately leads to the development of osteoarthritis. Stem cell-based therapy has been proposed for the treatment of osteoarthritis (OA) and induced pluripotent stem cells (iPSCs) are becoming a promising stem cell source.

**Results:**

Three steps were developed to differentiate human iPSCs into chondrocytes which were transplanted into rat OA models induced by monosodium iodoacetate (MIA). After 6 days embryonic body (EB) formation and 2 weeks differentiation, the gene and protein expression of Col2A1, GAG and Sox9 has significantly increased compare to undifferentiated hiPSCs. After 15 weeks transplantation, no immune responses were observed, micro-CT showed gradual engraftment and the improvement of subchondrol plate integrity, and histological examinations demonstrated articular cartilage matrix production.

**Conclusions:**

hiPSC could be an efficient and clinically translatable approach for cartilage tissue regeneration in OA cartilages.

## Background

The increasing prevalence of degenerative cartilage diseases, particularly osteoarthritis (OA), presents an important social and healthcare problem. OA could become the fourth leading cause of disability by the year 2020 [1]. OA is mediated by several pathogenic mechanisms, including enzymatic degradation of extracellular matrix, deficient new matrix formation, cell death, and abnormal activation and hypertrophic differentiation of cartilage cells [2]. The traditional therapeutic options for OA are pharmaceutical interventions and joint replacement surgery [3]. Methods for regenerating chondrocytes and cartilage tissue are expected to substitute or supplement conventional therapies for such diseases. In this respect, the use of stem cells in combination with growth factors and scaffolds are highly considered as an ideal option for articular cartilage (AC) regeneration [4].

To date, AC regeneration and cartilaginous tissue engineering research has focused largely on the use of autologous chondrocytes and mesenchymal stem cells (MSCs) as cell resources. However, for autologous chondrocyte, donor site morbidity is a challenge [5]. Bone marrow MSCs (BMSCs) possess limited proliferation capability and decreased differentiation potential with increasing donor age [6]. Moreover, the invasive procedure required to harvest BMSCs presents another hurdle to widespread clinical application. Adipose derived stem cells are more easily harvested, but its differentiation potency is not as strong as embryonic stem cells. Generation of induced pluripotent stem cell (iPSC) offers an alternative cell source for regenerative medicine. Treatments of neural and cardiovascular disease models with iPS cell transplantation have already been reported [7–9]. Compared to other fields, the research for AC regeneration using iPS cells has just begun.

Human iPSCs (hiPSC) established from autogenous cells exhibit proliferation capability and pluripotency similar to those of human embryonic stem cells (hESCs), but no immune rejection and ethical problems. Moreover, to reduce the risk of tumorigenicity, new methods for generating iPSCs without viral vectors have been developed [10, 11]. Therefore, hiPSCs are viewed as a promising new tool for regenerative medicine. hiPSCs have been reported to generate cartilaginous tissue in teratoma in vivo [12, 13], but limited data exists at present regarding the in vitro chondrogenic differentiation of hiPSCs. A reproducible method for in vitro chondrogenic differentiation of hiPSCs hasn’t been established. Teramura et al. reported mouse iPSC-derived embryonic body (iPS-EB) derived cells expressed surface markers similar to MSCs, these cells could differentiate toward cartilage using TGF -β3 and BMP-2 [14]. Treatment of EBs with all trans-retinoic acid followed by TGF -β3 and BMP-2 could also induce chondrogenesis [15]. In terms of disease-specific iPS cells, human OA chondrocyte-derived iPS cells have been established and showed chondrogenic potential using EB formation or co-culture with chondrocytes [1, 16]. Koyama used a multistep culture method to differentiate hiPSCs into chondrocytes, about 70 % hiPSCs expressed type II collagen and aggrecan [17]. All these studies suggested that iPSC may be a potential alternative cell source for articular cartilage regeneration. The major drawback in the use of iPSCs for tissue engineering is the difficulty in obtaining a uniform interest cell population, which creates the danger of teratoma formation from undifferentiated cells [18]. Another drawback is the very low yield of the cells, together with the fact that they do not emerge in culture until 3 weeks after transduction [19]. All these caused the application obstacle of iPSC in tissue engineering.

In this study, we have successfully differentiated iPS cells into chondrocytes in vitro in a simple way with a high differentiation ratio, after transplantation of iPS derived chondrocytes into MIA induced OA model, we can see the repairation of knee lesion, and therapeutic effects can be observed from the improvement of knee function.

## Methods

### Cell culture and differentiation

The study was approved by Medical Ethical Committee and Animal Ethical and Welfare Committee of Shenzhen University. All patients provided written informed consent.

hiPSC was generated by introducing four reprogramming factors (Oct3/4, Sox2, Klf4, and c-Myc) into dermal fibroblasts from the OA woman (51 years old, Kellgren & Lawrence scoring III), and characterized by Peking Cellapy Biotechnological company. Three of the identified iPS clone were used in this study and hiPSCs were maintained with PSCeasy medium (Cellapy Bio, China), hiPSCs after passage 20 from three clones were used for the differentiation experiments. iPSC from one of the clones were used for transplantation. For EB formation, hiPSC colonies were harvested by treating with 0.5 mg/mL Dispase, and then plated onto suspension culture dishes, where they were allowed to aggregate in a maintenance medium (DMEM/F12 supplemented with 10 % FBS, 100U/mL penicillin, and 100 μg/mL streptomycin). After 5 days as a suspension culture, EB media were changed for chondrogenic media (DMEM/F12 supplemented with 10 % FBS, 1%ITS, 50 μg/mL ascorbic acid 2-phosphate, 100 mg/mL sodium pyruvate, 40 μg/mL L-proline, 100nM dexamethasone, 10 ng/mL transforming growth factor-β1 (TGF- β1), 100U/mL penicillin, and 100 μg/mL streptomycin) and cultivated for another 2 days. Then five EBs were transferred to gelatin coated dish with chondrogenic media, after 1 and 2 weeks differentiation, cells sprouted from EBs were examined, and the cells which differentiated for 2 weeks were harvested for cell transplantation. EBs cultivated in DMEM which contained only 10 % FBS, 100 U/ml penicillin, and 100 μg/ml streptomycin used as control. All materials in the media are purchased from Invitrogen.

### Histological analysis and immunofluorescence assay

After cells were sprouted from EB and differentiated for 1 or 2 weeks, we fixed differentiated cells and undifferentiated iPSCs in 4 % paraformaldehyde (pH 7.4, Sigma-Aldrich) at room temperature, and stained with toluidine blue. For the immunofluorescence assay, undifferentiated iPSCs and their differentiated derivatives were fixed in cold acetone for 30 min at 4 °C, then cells were rinsed with PBS three times and blocked in 10 % normal goat serum for 20 min. Samples were incubated with primary antibodies (Anti-CollagenII, Abcom) overnight at 4 °C, then rinsed with PBS three times and incubated with Alexa Fluor 594 Donkey-conjugated secondary antibodies (Invitrogen) for 2 h at room temperature. Cells were rinsed with PBS and mounted in aqueous mounting medium containing the nuclear counterstain DAPI (4,6 diamidino-2-phenylindole; Sigma-Aldrich). Images were captured using an IX-71 fluorescence microscope (Olympus, Japan).

### Real-time reverse transcription–polymerase chain reaction

Total RNA was extracted from cell pellets (5 × 10^5^cells/group) with Trizol (Qiagen). To ensure the complete removal of DNA, we included a 15-min DNase I (Qiagen) treatment before the washing step. First-strand cDNA synthesis was performed using PrimeScript™ RT reagent Kit with gDNA Eraser (TaKaRa) with random hexamers. Real-time reverse transcription–polymerase chain reaction (RT-PCR) was performed with SYBR Premix Ex Taq™ II (Tli RNaseH Plus) (TaKaRa). Complementary DNA was mixed with TaqMan Universal PCR Master Mix (Applied BioSystems) and TaqMan Gene Expression Assay (Applied BioSystems). All RNA samples were titrated to yield equal amplification of β-actin as an internal normalization control, and the expression level of each mRNA was calculated using the 2^-∆∆CT^ method. Reactions for each sample were performed in triplicate. After an initial denaturation step (95 °C for 30s), amplification was performed for 40 cycles (5 s denaturation at 95 °C and 30s extension at 60 °C). Table 1 showed the primer sequences we used in PCR.

**Table 1:** Primer sequences of sepecific genes

Primer name	Sequence(5' to 3')	
Sox 9	F	GGAGATGAAATCTGTTCTGGGAATG
	R	TGAAGGTTATCTGCTGGTGTTCTGA
ACAN	F	TCTACCGCTGCGAGGTGA
	R	TGTAATGGAACACGATGCCTTT
Col2a1	F	GGAAGAGTGGAGACTACTGGATTGAC
	R	TCCATGTTGCAGAAAACCTTCA
Nanog	F	ACAACTGGCCGAAGAATAGCA
	R	GGTTCCCAGTCGGGTTCAC
Oct 4	F	GGAGGAAGCTGACAACAATGAAA
	R	GGCCTGCACGAGGGTTT
β-actin	F	CGAGAAGATGACCCAGATCATG
	R	ACAGCCTGGATAGCAACGTACA

### OA induction and cell transplantation

Sprague Dawley rats (weight 200–230 g) were housed under standard laboratory conditions (in a temperature controlled (21 ± 1 °C) room with a normal 12-h light/dark cycle). Animals retained full mobility and continued to grow normally. For induction of MIA-induced arthritis, rats were anesthetized with 2 % Pentobarbital Sodium and given a percutaneous single intra-articular injection of 0.5 mg of monosodium iodoacetate (MIA; Sigma) in right knee, or saline vehicle through left knee (*N* = 3). MIA was dissolved in physiologic saline and administered in a volume of 25 *μ*l using a 26-gauge, 0.5-inch needle. The left contra-lateral knee was used as a behavioral and histological control. The hind limbs of all the rats were imaged with high resolution in vivo micro-CT at 1 and 5 weeks post-MIA injection. After 1 week MIA injection, iPSCs and differentiated iPSCs were collected, adjusted cell density as 1 × 10^6^cells/ml, then 500 *μ*l cell suspension were injected into right knee. After 15 weeks transplantation, all the knees were imaged with micro-CT and examined by sections. Total 13 SD rats were used in this study, three for control and MIA injection, 10 for MIA injection and cell transplantation (five for iPS cells and five for iPS-derived chondrocytes, right knee for MIA injection, left knee for cell transplantation after MIA injection, total knee sample for each group is 10). The animal handling and experimental procedures outlined in this study were carried out in accordance with Animal Ethical and Welfare Committee of Shenzhen University and The Institute of Medical and Veterinary Science Animal Ethics Committee.

### In vivo micro-computed tomography (micro-CT) imaging

In vivo micro-CT imaging was performed using a bench-top cone-beam type in vivo animal scanner (Skyscan model 1076, Skyscan, Kontich, Belgium). Each rat was anesthetized with Pentobarbital Sodium and placed in the scanner bed in a supine position. The MIA-injected knees and the contralateral control knees were scanned separately. During each scan only the knee for image data acquisition was irradiated, while the contralateral limb and the rest of the body were lead shielded from radiation. The hind limb of the rat was secured into a customized leg fixative device consisting of a cylindrical plastic holder fitted to a polystyrene tube. This allowed positioning of the hind limb close to the central scanner axis, preventing any movement of the limb during scanning. The total scan time for each limb was 20 min during which the rat was under anesthesia. The scans were performed using the following scanner settings: X-ray source voltage 60 kVp, current 100 μA, a 1-mm thick aluminum filter to reduce beam-hardening artefact, one frame averaging. The pixel size was 9 μm, the exposure time was 1.1 s, and the rotation step was 0.8°, with a complete rotation over 180°. The cross-sectional images were reconstructed using a filtered back-projection algorithm (NRecon, V 1.4.4, Skyscan, Kontich, Belgium). For each scan, a stack of 1800 cross-sections was reconstructed, centered over the knee-joint (total reconstructed height about 16 mm), with an interslice distance of one pixel (9 μm). The reconstructed images were of 1500 × 1500 pixels each, 9 μm pixel size, and were stored as 8-bit images (256 grey levels).

### Histological analysis

After imaging, the animals were sacrificed and the each knee was dissected aseptically, and fixed in 4 % paraformalin, then decalcified in EDTA, which was changed every 5 days. The decalcified knee was cut in the mid-sagital plane, and paraffin-embedded. Serial knee sections of exact 5 μm thickness from the middle part of the knee were obtained to prepare slides. Rat knee joints were stained with hematoxylin and eosin (H&E) to assess general morphology and neovascularization. For immuno-histochemistry, Anti-CollagenIIprimary antibody (Abcam) was used in 1:500 dilution. All samples from both knees were stained, and examined independently by two observers. Sections were stained for GAGs with 0.5 % Safranin-O and 0.2 % Fast Green counterstain. For each sample, three sagittal sections were used for thickness histomorphometry analysis and digital images of each section were captured.

### Statistical analysis

Data are presented as mean ± standard deviation. The comparisons among different groups were performed using multiple–factorial analysis of variance (ANOVA). When ANOVA testing indicated overall significance of main effects and without interaction between them, the difference between individual time points and sites was assessed by post hoc tests. The level of significance was set at P < 0.05. All data analyses were performed using SPSS 15.0 analysis software (SPSS Inc).

## Results

### Morphological changes of hiPSCs during differentiation

**Fig. 1:**
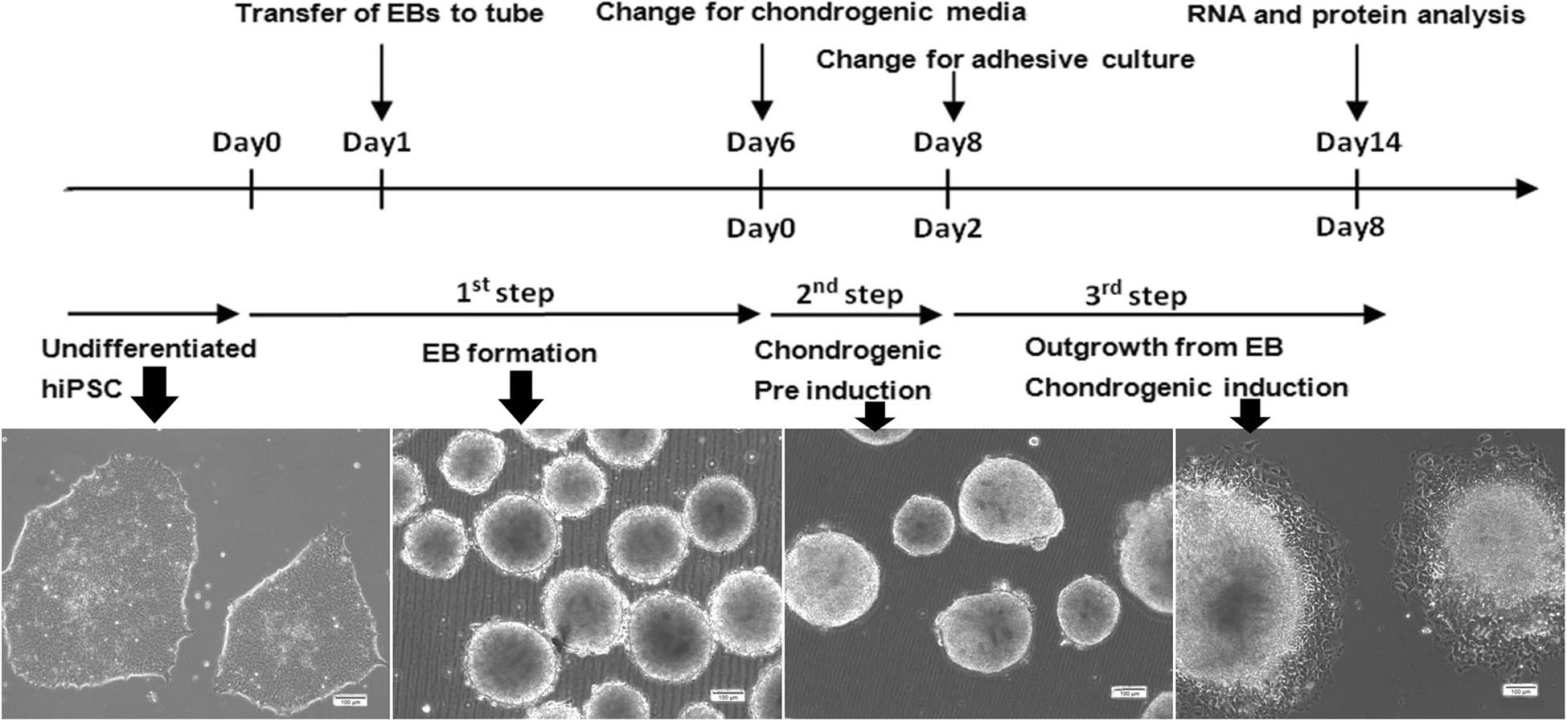
Schematic diagram of the culture protocol for chondrogenic differentiation of hiPSCs. The differentiation culture protocol consists of 3 steps: (1) Embryoid body (EB) formation in suspension culture dishes; (2) Pre induction of EB in the chondrogenic induction medium; (3) Cell outgrowth from EBs on gelatin-coated dishes in the chondrogenic induction medium. During differentiation, histological and gene expression analyses were performed at days 7 and 14. Scale bar: 100 μm

We used a three step culture method combining spontaneous differentiation via EB formation, pre induction in EB suspension culture, cell outgrowth from EBs on culture dishes (Fig. 1). We first made undifferentiated hiPSCs into EBs. hiPSCs contained in EBs retained pluripotency in vitro. Before proceeding to adhesive culture, we included a pre induction with chondrogenic media in EB suspension culture. After 2 days pre induction, EBs were transferred to gelatin coated culture dish, cells were sprouted from EB and differentiated in chondrogenic media for another 1 or 2 weeks.

Before differentiation, hiPSCs in monolayer culture were small and fibroblast-like,and grew as colon while cultured on matrigel, we chose no differentiation colons with similar size for EB formation. After 5 days suspension culture, we replaced the EB media for chondrogenic media as pre induction. During pre induction, EBs exhibited better morphology and less internal necrosis. When EBs were cultured in gelatin coated culture dish, some large spherical cells with chondrocyte-like morphology sprout from EBs.

### Immunohistochemical analysis of chondrogenic differentiation markers

**Fig. 2:**
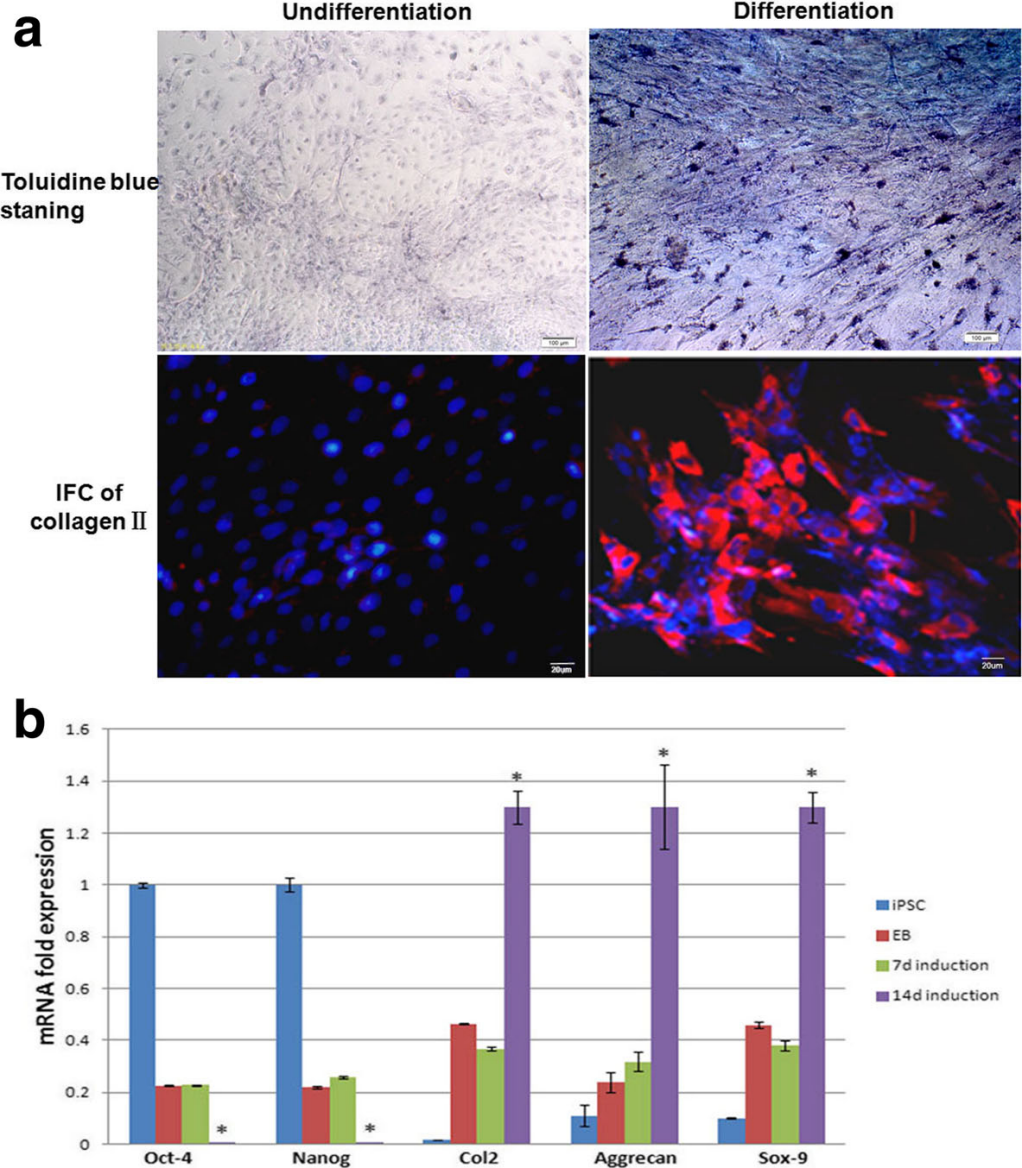
Examination of chondrogenic markers after differentiation. After EBs were differentiated for 2 weeks, staining with Toluidine blue revealed spherical cell morphology and interstitium, type II collagen was also detected after differentiation (**a**). The expression of chondrogenic differentiation markers was also analyzed by real-time PCR (**b**). Gene expression is normalized to that of beta–actin. Differentiated hiPSCs in monolayer culture exhibited high expression of COL2A1, ACAN and SOX9 compare with undifferentiated iPSCs. The expression was increased significantly at later stages (day 14). *Significant change relative to control (*n* = 4). Data are shown as the mean ± SD

After 2 weeks induction, the entire EB with sprouted cells were intensely stained with Toluidine blue (Fig. 2a), suggesting maturation of cartilaginous extracellular matrix. EBs cultivated in DMEM as control showed Toluidine blue negative staining. To confirm the chondrogenic differentiation capability of hiPSCs, we detected type II collagen expression (Fig. 2a). Cells in differentiated EBs exhibited strong positive staining for type II collagen. EBs cultured with DMEM did not exhibit cell morphological change or the expression of chondrogenic markers.

After confirming the expression of chondrogenic differentiation marker by immunohistochemistry in both differentiated and undifferentiated hiPSC, we next examined the expression of chondrogenic markers at the mRNA level (Fig. 2b). We analyzed both chondrogenic differentiation markers and ES cell markers expression by real-time RT-PCR after 7 and 14 days differentiation. COL2A1, ACAN and SOX9 expression was also present in undifferentiated EBs, however, after 2 weeks for differentiation, the expression of COL2A1, ACAN and SOX9 was dramatically upregulated. Coordinarily, ES cell markers, Oct4 and Nanog expression was decreased significantly compare to undifferentiated EBs and iPSCs. However, it seems the expressions of both kinds of markers are similar between undifferentiated EBs and the EBs differentiated for 14 days.

### Bone histomorphometric changes and subchondral trabecular bone

**Fig. 3:**
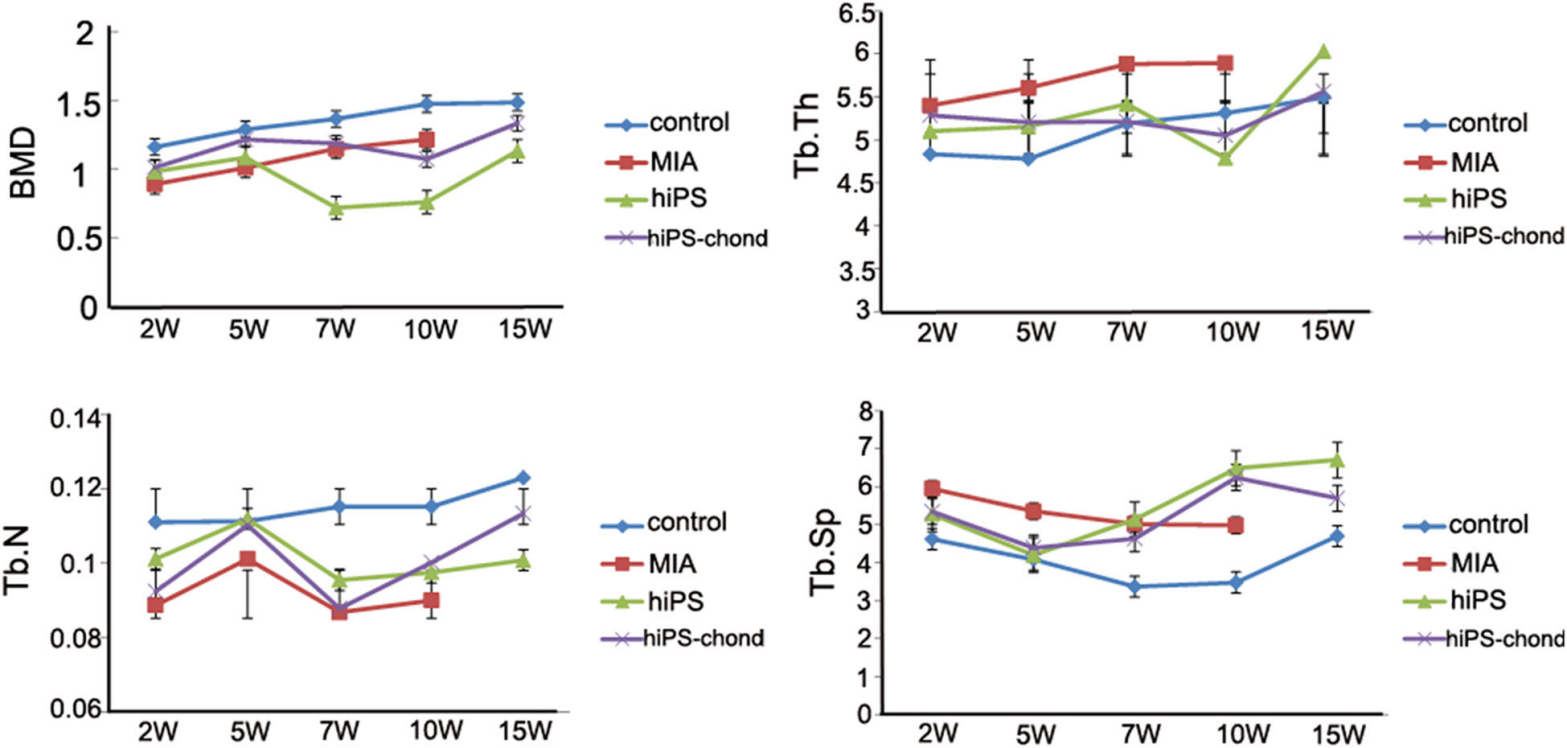
Plots of morphometric parameters of subchondral trabecular bone determined by micro-CT at 2, 5, 7, 10 and 15 weeks post-MIA injection. MIA-injected rat almost couldn’t move after 10 weeks, and it’s too fat to do the CT examine, so no data can be get at 15 weeks in MIA group. Error bars = SD. MIA, monosodium iodoacetate; BMD, Bone Mineral Density ; Tb.Th, trabecular thickness; Tb.N, trabecular number; Tb.Sp, trabecular separation

The microstructure of subchondrol bone change with OA, to assess the change of bone mass and bone trabecula, microCT was used to observe the changes of bone mineral density (BMD), Tb.Th, Tb.N and Tb.Sp at 2, 5, 7, 10 and 15 weeks. There was a significant increase in total BMD, Tb.Th and Tb.N in the tibial subchondral bone of both the MIA injected knee and the control knee over time (Fig. 3). This was due to the natural growth of the animal. The total Tb.Sp of both the control tibia and the tibia of the MIA-injected knee were significantly decreased at 15 weeks compared to 2 weeks (Fig. 3).

The BMD and Tb.N were significantly lower in the MIA-injected knee compared to the control knee in all the three tibial compartments. In the 5 weeks after cell injection, there was a slightly increase of BMD, at 7 weeks, the BMD was significantly low and maybe the osteoporosis was appeared. However, the BMD was increased quickly after 10 weeks, and it was even close to the control knee, the possible reason is endochondral ossification. At 5 weeks, the Tb.N was suddenly increased significantly, bone trabecules were broken in subchondral bone may cause the higher Tb.N. At 15 weeks, the Tb.N did not differ significantly between the tibia of the cell-injected knee and control knee.

The Tb.Th and Tb.Sp were increased in the MIA-injected knee compared to the control knee at all the time points. After cell injection, the values of Tb.Th were decreased and almost close to the control knee at 7 weeks. In the 5 weeks of cell injection, although there was a slightly decrease of Tb.Sp, it were increased quickly in the following weeks. After 15 weeks, the knee injected with hiPSC derived chondrocyte, the values of BMD, Tb.Th, Tb.N and Tb.Sp were much more close to the control knee, compared with the knee injected with hiPSC.

### Qualitative subchondral bone changes

**Fig. 4:**
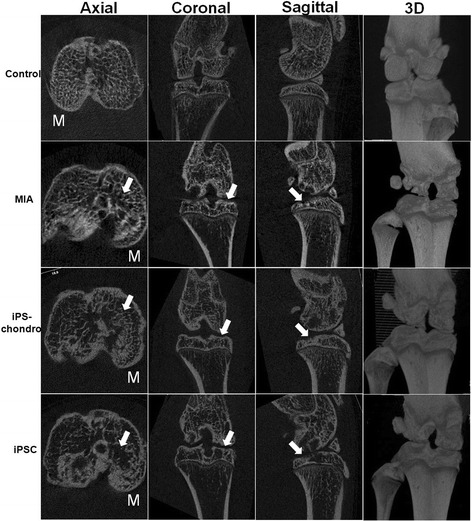
Micro-CT images of tibia at 15 weeks post injection. Three-dimensional surface rendering obtained from micro-CT images of a control knee (Control), a MIA-injected knee (MIA), iPS cells transplanted knee (iPSC) and iPS derived chondrocytes transplanted knee (iPS-chondro) at 15 weeks after injection. The control knee maintained the subchondral plate integrity with a smooth contour. The MIA-injected knee showed erosion and pitting of the tibial subchondral plate, which was more severe in the medial tibial plateau. Micro-CT, micro-computed tomography; MIA, monosodium iodoacetate; M, medial tibial plateau

All the rats showed pathological subchondral bone changes in the MIA-injected knee, whereas the contralateral control knee showed no OA-like changes in the tibial subchondral bone microarchitecture throughout the study duration (15 weeks). Morphologic evaluation of the MIA-injected knee over time showed sclerosis in the tibial subchondral bone at 15 weeks after MIA injection, particularly in the medial tibial condyle (Fig. 4). The tibial subchondral plate of the MIA-injected knee was breached in focal areas, these breaches were confirmed by histology. From the micro-CT images, empty spaces were observed in the medial tibial subchondral bone of the MIA-injected knee at 15 weeks. These empty spaces were confirmed as cysts from the histology sections. The porosity of the tibial subchondral plate was significantly increased in the MIA-injected knee compared to the control knee in all the three compartments analyzed (P < 0.01). Three dimensional surface rendering of the MIA-injected knee showed erosion and pitting of the tibial subchondral plate, which was more severe in the medial tibial plateau, whereas the control knee maintained the subchondral plate integrity. After one week MIA injection, we transplanted iPS cells or iPS derived chondrocytes into articulation, at 15 weeks, iPS cells and iPS derived chondrocytes transplanted knee didn’t show any significant difference, however, compared with MIA-injected knee, no obvious breaches and empty spaces were observed in the tibial subchondral bone, the porosity of the tibial subchondral plate was slightly decreased compare with MIA-injected knee (Fig. 4).

### Macroscopy

**Fig. 5:**
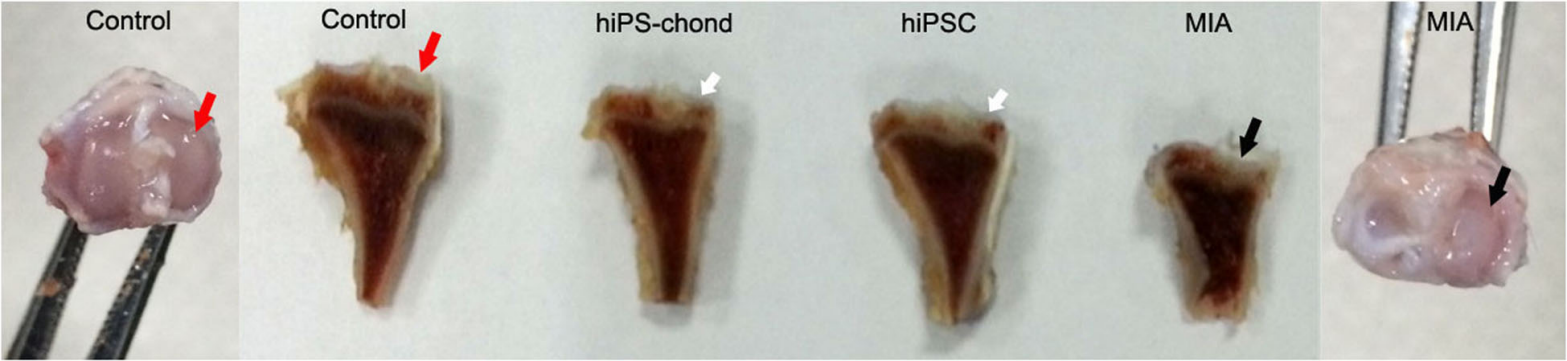
Macroscopic images of tibia at 15 weeks post injection. The control tibia had no cartilage lesions on the medial compartment and lateral compartment of the tibial plateau (*red arrow*), whereas the MIA injected tibia had severe cartilage lesions on the medial tibial plateau (*black arrow*). After cell transplantation, the repair was obviously observed in iPS derived chondrocytes transplanted knee (*white arrow*). MIA, monosodium iodoacetate, all the arrow point to medial tibial plateau

All the rats developed cartilage lesions in the tibia of MIA-injected knees. After 15 weeks cell injection, there was no significant difference compared with MIA-injected knees. The cartilage plates were easily peel off from the joint, however, after MIA and cell injection, the cartilage plates were sticked to the joint and hard to peel off. The tibial cartilage of the MIA-injected and cell injected knee all showed focal lesions with discoloration indicating cartilage degradation (Fig. 5, black arrow). This was more prominent in the central region of the medial tibial plateau, whereas cartilage degradation remained at a moderate stage on the lateral side. Macroscopically, the control tibia showed no cartilage lesions in the medial and lateral tibial plateau (Fig. 5, red arrow). Cartilages were repaired after the transplantation of iPSCs and iPS derived chondrocytes. hiPS derived chondrocytes seems have stronger repair ability than hiPSCs (Fig. 5, white arrow).

### Histology

**Fig. 6:**
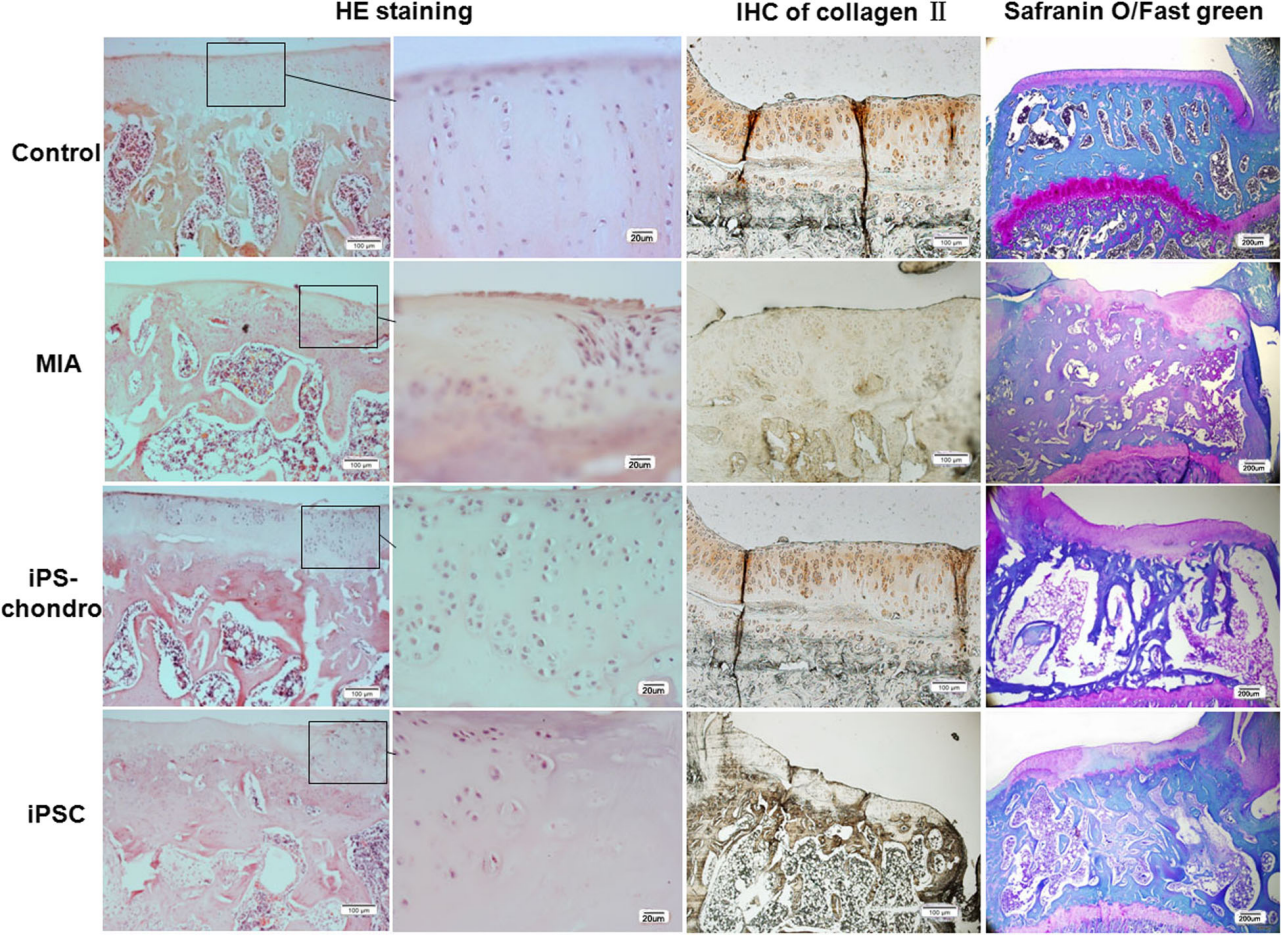
Histological analysis of knee sections. HE staining, Immunostaining of collagen II, Safranin O/Fast green staining were did to test the repairment of iPSCs. MIA-injected knee (MIA) showed obvious cartilage damage compare with normal knee (Control), while transplantation of iPS cells or iPS derived chondrocytes, the cartilage was repaired, and iPS derived chondrocytes (iPS-chondro) seems have stronger repair ability than iPS cells (iPSC)

The articular cartilage from control knee exhibited typically four intact cell layers including a superficial, transitional, deep radial, and deep calcified zone, and uniformly-distributed proteoglycan as the main extracellular matrix component (Fig. 6). However, all the rats showed articular cartilage degradation in the tibia of the MIA-injected knee. At 15 weeks, the subchondral plate was breached in focal areas with cartilage damage. The sections showed OA-like features such as surface discontinuity, loss of proteoglycans, disorientation of chondrocytes and subchondral bone sclerosis. After transplantation of iPS derived chondrocytes, cartilage surface was continue, proteoglycan was increased compare with MIA-injected knee, and appeared proliferating chondrocytes suggesting cartilage repair. While after iPS cells transplantation, the repair was not so obvious (Fig. 6).

Immunostaining of the neo-cartilage for collagen II also indicate the repair of iPS derived chondrocytes (Fig. 6). The MIA cartilage showed the less collagen compare with control cartilage, while after injection of iPSCs and iPS derived chondrocytes, collagen II increased compare with MIA cartilage, iPS derived chondrocytes injection can produce more collagen II compare with iPSCs injection, which indicate that iPS derived chondrocytes have better repair effect than iPSCs only. However, the amount of collagen IIin cartilage after iPSCs injection still couldn’t reach to that of control cartilage.

The images of tibial articular cartilage sections stained with Safranin O-Fast green are shown in Fig. 6. The MIA cartilage displayed extensive proteoglycan loss, which was not observed in the saline injected contralateral control. After tansplantation of iPSCs, it showed localized degeneration with regional thickening of the medial tibial plateau, oblique fissures within the superficial bone were still exist, subchondral bone resorption pit, remodeling and thickening subchondral bone can be observed. However, the restore efficiency was much better in iPS-chondrocyte group than the iPSC group.

## Discussion

Articular cartilage tissue is hard to regenerate after injury because of the innate avascularity, low cellularity and low cell turnover. Therefore, the development of treatments allowing successful regeneration of cartilage lesions to avoid further degeneration of focal articular defects represents a major challenge [20]. Researchers tried to find good methods to replace or repair diseased or damaged cartilage tissue [21–23]. Cell regeneration seems a good way to regenerate cartilage tissue, and it has been driven by discovery of iPSCs. iPSCs can proliferate and differentiate into a variety of different lineages, and it can be applied to personalized therapies, which provide an intriguing alternative to the use of stem cells in regenerative medicine [24, 25].

The differentiation efficiency of iPSC into chondrocyte became the main obstacle in clinic use. There are two methods which have been used to induce the differentiation of stem cells into chondrocytes: application of growth factors in medium and co-culture with chondrocytes. However, both culture with a conditioned medium and co-culture with other cells lack reproducibility and efficiency. Many attempts have been made to induce chondrocytes from ESCs in vitro by pellet or micromass culture with co culturing system [26, 27] or limb bud progenitor cells from a developing embryo, culture with a conditioned medium, genetic manipulation, and use of biomaterials [28, 29]. In our study, we made EBs from iPSCs, the high-density 3D microenvironment is thought to facilitate cell–cell and cell–matrix interactions, and therefore mesenchymal condensation occurs before chondrogenic induction. After EB formation, we did 2 days pre induction to differentiate chondrocytes in 3D microenvironment, which mimic in vivo limb development. When differentiate chondrocytes in a two dimensional (2D) EB direct-plating outgrowth system, cells change their morphology, express chondrogenic differentiation markers, and produce an extracellular matrix that contains acidic proteoglycans, which stain positive for Toluidine blue, the positive area and intensity of Toluidine blue staining, which indicated the existence of acidic proteoglycans. The chondrogenic transcription factor Sox9 is required for precartilage condensation and directly regulates the transcription of type II collagen and aggrecan [30–32], Type II collagen is an early chondrogenic differentiation marker, and aggrecan is a major sulfated proteoglycan of the cartilage matrix and a highly specific marker of differentiated chondrocytes [32, 33]. From the genetic and protein expression studies, we can see that Sox9, aggrecan and collagen II expression increased significantly after differentiation, which indicate the successful differentiation of iPSCs into chondrocytes. Previous investigations, whatever coculture system or conditional medium, expression level of chondrogenic markers was low, and immunofluorescence did not clearly reveal morphological characteristics of chondrocytes [34], a discrepancy between gene expression and protein expression of type II collagen has been reported, which means translation might be destabilized in the culture conditions, and cells in pellets are still immature chondrocytes, and do not produce enough collagen to be detected by immunohistochemistry besides, it need multiple steps and a long time to show the chondrocyte specific markers [17]. In our differentiation system, we developed an efficient, simple and reproducible system to differentiate iPSCs into chondrocytes, the total process from iPSC to chondrocyte is just 2 to 3 weeks. During induction, all the cells spread out from EB can get enough TGF for differentiation, besides, preinduction might also improve maturation of chondrocytes in our method, resulting in enhanced expression of type II collagen and aggrecan protein.

To validate the repair capacity of human iPSCs for OA knee, in vivo study was performed. Many investigators have shown that intra-articular injection of MIA, which is an inhibitor of glycolysis that disrupts metabolism in chondrocytes, causes joint tissue damage that may mimic clinical OA in patients [35–37] The MIA model leads to global degenerative changes, which is a quick, easy and reproducible OA model, and animals exhibit signs of OA related pain. So in this study, we used the MIA-induced OA rat model to pursue iPSC therapeutic efficiency for OA knee. Using this verified animal model, we examined the therapeutic effects of iPSCs and iPS derived chondrocytes.

After iPS cell transplantation, we found some areas of the articular cartilage were still intact, the thickness of the subchondral plate became thinner compared with MIA-injected knee, iPS derived chondrocytes transplanted articular cartilage have thicker subchondral bone compare with that of iPSCs transplantation. The subchondral bone and articular cartilage act as a functional unit in the joint [38]. In human osteoarthritis joints, the subchondral plates become significantly thicker relative to that of healthy subjects [39, 40]. The subchondral bone was modeled post surgery in MIA-induced OA models and their thickness became thinner, which is similar with the change of subchondral bone in human OA.

The obvious cartilage tissue formation generated from iPSCs was observed from histological examination at 15 weeks. Because of low immunity, no immune responses were observed after iPSC transplantation. The observation suggests that iPSCs and iPS derived chondrocytes have therapeutic effects, and partial differentiated iPSCs may even have stronger therapeutic effects than that of pure iPSCs. Compare with MIA cartilage, there appeared more collagen and aggrecan, which indicate that iPSC injection can differentiate more chondrocytes and produce more collagen and aggrecan to repair damaged cartilage. Several studies also indicated that combination of mesenchymal stem cells with chondrocytes had better therapeutic effects than mesenchymal stem cells only [41]. The reason could be that partially differentiated iPSCs have more chondrogenic potential than undifferentiated iPSCs in vivo chondrogenic microenvironment. The therapeutic effects were also identified by the activity of rats. After OA injection, rats moved slowly and became fat, and nearly cannot move at all at 10 weeks. However, after cell transplantation, rats were thinner and moved faster than OA rats, which indicate the improved joint functionality and reduced lameness.

Even though we observed improved joint functionality and reduced lameness after cell transplantation, but the rats still have dyskinesia, and the joint didn’t recover their function completely. The direct injection of stem cells to the joint can boost the normality limited repair and limit destructive processes, it may be only suitable for the early-stage OA, further work is needed to focus on stem cell based cartilage tissue engineering strategies for end-stage OA.

## Conclusions

In summary, we developed an efficient, simple and reproducible system to differentiate iPSCs into chondrocytes with a high differentiation ratio, the total process from iPSC to chondrocyte is just 2 to 3 weeks. After transplantation of iPS derived chondrocytes into MIA induced OA model, the knee lesion was obviously repaired, and therapeutic effects can be observed from the improvement of knee function. The changes in cartilage and subchondral trabecular bone structure observed in the MIA rat model before and after transplantation reveal the therapeutic effects of iPS derived chondrocytes, at the same time, micro-CT and histological examination showed that subchondral trabecular bone structure didn’t recover completely, cartilage tissue engineering may be needed for the serious OA therapy.

## Abbreviations

3D: Three dimensional.
AC: Articular cartilage
BMD: Bone mineral density
BV: Bone volume
BV/TV: Bone volume fraction
hESC: Human embryonic stem cell
iPSC: Induced pluripotent stem cells
MIA: Monosodium iodoacetate
Micro-CT: Micro computed tomography
MSC: Mesenchymal stem cell
OA: Osteoarthritis
Tb.N: Trabecular number
Tb.Sp: Trabecular separation
Tb.Th: Trabecular thickness
